# Illumina microRNA profiles reveal the involvement of miR397a in *Citrus* adaptation to long-term boron toxicity via modulating secondary cell-wall biosynthesis

**DOI:** 10.1038/srep22900

**Published:** 2016-03-10

**Authors:** Jing-Hao Huang, Yi-Ping Qi, Shou-Xing Wen, Peng Guo, Xiao-Min Chen, Li-Song Chen

**Affiliations:** 1 Institute of Plant Nutritional Physiology and Molecular Biology, Fujian Agriculture and Forestry University, Fuzhou 350002, China; 2 Pomological Institute, Fujian Academy of Agricultural Sciences, Fuzhou 350013, China; 3 College of Life Sciences, Fujian Agriculture and Forestry University, Fuzhou 350002, China; 4 Institute of Materia Medica, Fujian Academy of Medical Sciences, Fuzhou 350001, China; 5 College of Resource and Environmental Science, Fujian Agriculture and Forestry University,Fuzhou 350002, China; 6 Fujian Key Laboratory for Plant Molecular and Cell Biology, Fujian Agriculture and Forestry University, Fuzhou 350002, China

## Abstract

The mechanisms underlying tolerance to B-toxicity in plants are still controversial. Our previous studies indicated that B-toxicity is mainly limited to leaves in *Citrus* and that alternations of cell-wall structure in vascular bundles are involved in tolerance to B-toxicity. Here, miRNAs and their expression patterns were first identified in B-treated *Citrus sinensis* (tolerant) and *C. grandis* (intolerant) leaves via high-throughput sequencing. Candidate miRNAs were then verified with molecular and anatomical approaches. The results showed that 51 miRNAs in *C. grandis* and 20 miRNAs in *C. sinensis* were differentially expressed after B-toxic treatment. MiR395a and miR397a were the most significantly up-regulated miRNAs in B-toxic *C. grandis* leaves, but both were down-regulated in B-toxic *C. sinensis* leaves. Four auxin response factor genes and two laccase (LAC) genes were confirmed through 5′-RACE to be real targets of miR160a and miR397a, respectively. Up-regulation of *LAC4* resulted in secondary deposition of cell-wall polysaccharides in vessel elements of *C. sinensis*, whereas down-regulation of both *LAC17* and *LAC4*, led to poorly developed vessel elements in *C. grandis*. Our findings demonstrated that miR397a plays a pivotal role in woody *Citrus* tolerance to B-toxicity by targeting *LAC17* and *LAC4*, both of which are responsible for secondary cell-wall synthesis.

Boron (B), an essential micronutrient, plays a crucial role in plant growth and development as well as sexual reproduction^1^,^2^. It is involved in the formation of the primary cell wall^3^, membrane integrity^4^,^5^ and cell-wall structure^6^ and participates in diverse physiological and biochemical processes, including cell-to-cell signalling^7^, secondary metabolism^8^, and gene expression^9^, in higher plants. However, our knowledge of the exact role of B in intercellular processes is still limited.

Given that the concentration range from deficiency to toxicity is narrower for B than for any other plant essential nutrient^10^, B-deficiency^11^ and/or B-toxicity^12^,^13^ can easily be observed in diverse plant species, including *Citrus* species. B-deficiency can be corrected by applying B fertilizers. However, improper application of B fertilizer leads to B-toxicity^13^,^14^. In agricultural lands in different regions of the world, B-toxicity often occurs in B-rich soils or in soils where B accumulates due to a continual inflow of desalinated irrigation waters or industrial pollutants that are rich in B, resulting in a low yield and poor quality of many crops and horticultural plants^12^,^15^. However, the molecular basis of B-toxicity in *Arabidopsis thaliana* was not reported until recently, by Aquea *et al.*^16^. These authors found that B-toxicity induced the expression of genes related to ABA signalling but repressed the expression of genes encoding water transporters, triggering a water-stress response associated with root growth inhibition.

Plant tolerance to B-toxicity varies widely among species and/or cultivars^12^,^13^,^17^,^18^. However, the mechanisms underlying tolerance to B-toxicity in plants are still controversial. The most widely accepted theory, suggested for herbaceous plant species, is that there is a reduction of intercellular B levels through B efflux transporters. In barley (*Hordeum vulgare*), for instance, up-regulation of the B efflux transporter *BOR1* in the roots decreases the absorption of B from soils^15^,^19^,^20^, whereas up-regulation of *BOR2*, which is homologous to *BOR1*, in the leaves leads to transport of B out of symplasts and into apoplasts^17^,^18^. Similarly, down-regulation of another efflux transporter, nodulin-like intrinsic protein (NIP) in roots can also decrease B transport from roots to shoots, thus reducing B accumulation in aboveground plant parts^21^. However, a recent physiological study by Landi *et al.*^22^ indicated the involvement of photoprotection by foliar anthocyanins in the tolerance mechanisms of sweet basil (*Ocimum basilicum*) to B-toxicity.

In *Citrus*, B-toxicity mainly occurs in the leaves and has little phenotypic effect on the roots. Although similar total B levels have been observed in the leaves or roots of tolerant and intolerant species, free B levels are significantly lower in the leaves of tolerant species than in those of intolerant species^23^. Nevertheless, transcriptome^24^ and proteome analyses^25^ have not yet identified any differentially expressed genes homologous to either *BORs* or *NIPs* from B-toxic *Citrus* leaves or roots. Further anatomical studies showed that the effects of B-toxicity on leaf structure are mainly limited to leaf veins. B-toxicity results in intensive exocytosis, accompanied by cell-wall thickening in the phloem of tolerant *C. sinensis* but induces cell death of phloem tissue through autophagy in intolerant *C. grandis*^23^. Our previous studies suggested that other mechanisms of tolerance to B-toxicity might exist in woody *Citrus* plants.

MicroRNAs (miRNAs) are 21–24 nucleotide (nt) small RNAs generated from non-coding RNA genes. As important post-transcriptional regulators, miRNAs have been shown to negatively regulate gene expression by directing the slicing of target mRNAs in the central portion of complementary regions^26^ or through inhibition of target mRNA translation^27^,^28^. Evidence demonstrates that plant miRNAs play a crucial role in the regulation of plant growth and development as well as the response to biotic and abiotic stresses^29^,^30^,^31^,^32^. For example, in *A. thaliana*, miR399 has been experimentally proven to maintain phosphorus (P) homeostasis by regulating *UBC24* transcript levels^33^, whereas miR395 mediates the regulation of sulfate (S) accumulation and allocation by targeting *APS* and *SULTR2;1*^34^, respectively. Up-regulation of such miRNAs under P- or S-deficiency suggests that miRNAs are involved in the adaptive responses of plants to nutrient stress. Recently, two additional reports, by Ozhuner *et al.*^35^ for barley and Lu *et al.*^36^ for *Citrus*, indicated that a number of miRNAs are up- or down-regulated under either B-toxic or B-deficient conditions, suggesting that miRNA expression could also respond to micronutrient-related stress. Nevertheless, little information about B-toxicity-responsive miRNAs is available in woody plants.

With the objective of exploring the mechanism of tolerance to B-toxicity in woody plants, we report high-throughput sequencing data (Illumina) in addition to a bioinformatic analysis of small RNAs from the leaves of B-tolerant and B-intolerant *Citrus* species treated with different B levels. Predicted targets of the candidate miRNAs were then verified using modified 5′-RACE and quantified via qRT-PCR. On this basis, two validated target genes that function in cell-wall metabolism were anatomically verified. Our results indicated that miR397a plays a pivotal role in the tolerance of woody *Citrus* to B-toxicity by targeting genes that are responsible for secondary cell-wall synthesis.

## Results

### Plant growth

As we previously reported, in *C. grandis*, B-toxicity leads to progressive basal-to-top development of toxic symptoms in the leaves, beginning with tip yellowing, followed by marginal and interveinal chlorosis, and finally, senescence and abscission. However, no visible symptoms were observed in *C. sinensis* leaves under the same conditions, suggesting that *C. sinensis* is more tolerant to B-toxicity than *C. grandis*.

### Primary data analysis of sequences from small RNA libraries

The total numbers of raw reads generated from B-sufficient and B-toxic libraries via Solexa deep sequencing were 20,680,130 and 16,889,149 for *C. grandis* and 19,886,678 and 13,833,254 for *C. sinensis*, respectively. After removing adaptor sequences and filtering out low-quality and ‘N’-containing reads, 11,438,525 (55.31%) and 8,350,155 (49.44%) clean reads were retained from the B-sufficient and B-toxic libraries of *C. grandis* and 11,188,651 (56.26%) and 8,185,253 (59.17%) from those of *C. sinensis*, respectively. The numbers of unique clean reads generated from the libraries of the same species subjected to different B treatments were 1,988,267 (9.61%) and 1,350,479 (8.00%) in control and B-toxic *C. grandis* leaves and 1,593,866 (8.01%) and 1,214,103 (8.77%) in control and B-toxic *C. sinensis* leaves, respectively. The annotation of the sRNAs is shown in Table 1.

Based on the constructed library datasets, it was observed that the unique clean reads exhibited an uneven length distribution, with the majority (~85%) ranging from 18 to 24 nt in length (Supplementary Fig. 1). Among the unique clean reads, sRNAs of 21 nt were the most abundant, followed by those of 24, 20 and 22 nt. Further analysis revealed that on average, up to 8.23% of the 21 nt sequences, but only 1.25% of the 24 nt sequences, in the four libraries mapped to the *C. clementine* genome and were assigned to miRNAs, suggesting that 21 nt-long sRNAs are the most important miRNAs in *Citrus* plants. These results were consistent with previous reports by Song *et al.*^37^ for *C. trifoliate* and Yin *et al.*^38^ for soybean [*Glycine max* (L.) Merr.]. One of the important characteristic features of miRNAs is their initial 5′-uridine^39^. Here, 81.26 ± 3.11% of the 21 nt-long sRNAs in our four libraries started with a 5′ uridine, whereas 55.36 ± 3.82% of the 24 nt-long sRNAs began with a 5′ adenosine (Supplementary Fig. 2). Nevertheless, B-toxicity resulted in more 21 and 24 nt reads in *C. grandis* and fewer 20, 21 and 24 nt reads in *C. sinensis*.

### Identification of conserved and non-conserved miRNAs using sRNA libraries and bioinformatic analysis

**Figure 1:**
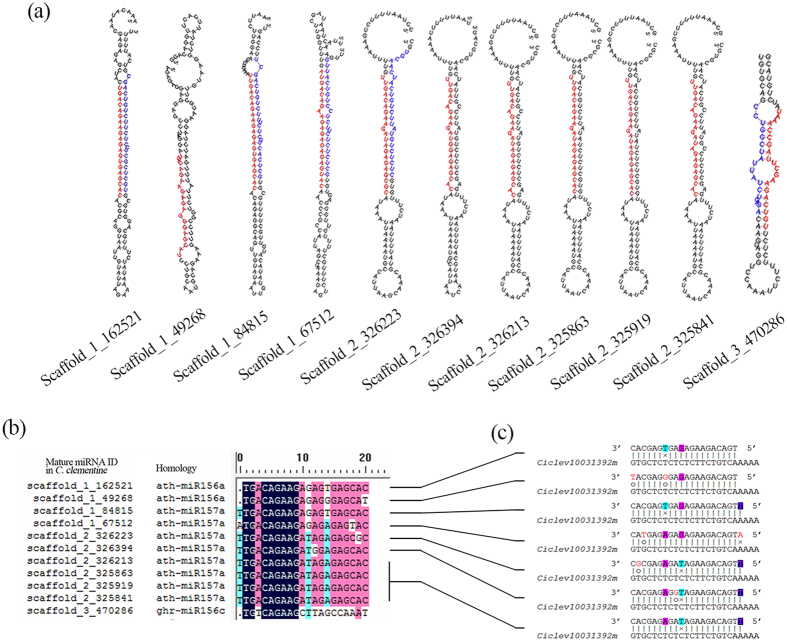
Bioinformatic analysis of miR156/157 members in *Citrus*. (**a**) Secondary structure of miR156/157 isoforms. miRNA precursor sequences were predicted using MTide (Zhang *et al.* 73) and folded by RNAfold (Hofacker, 2003). Positional information of each miRNA precursor refers to the start position on the respective chromosome (*C. clementine* genome v1.0). Cloned mature miRNA sequences were highlighted in red and miRNA* sequences in blue. (**b**) Alignment of mature miRNA isoforms using DNAMAN (version 7); ath, Arabidopsis; ghr, *Gossypium hirsutum*. (**c**) Polymorphism of miR156/157 members and their target (*Ciclev1001392m*) binding sites. miR-SNPs in miR156/157 are unlikely to change the target gene except for those in Scaffold_3_470286, which increased the mismatch positions, resulting in loss of its target. Watson-Crick pairing (vertical dashes), G:U wobble pairing (o) and mismatch (×) are indicated (the same below).

To identify known miRNAs, the sequences of the clean reads from the four sRNA libraries were aligned with known miRNAs from other plant species in miRBase 21. A total of 750,280 perfectly matched unique sequences were found to be orthologues of 221 reported miRNA families. These conserved miRNAs were assigned to 387 putative precursors, which showed dramatic variations in copy number ranging from 2 to 29,727, indicating a significant discrepancy in their accumulation in *Citrus* (Supplementary Dataset S1). It is worth mentioning that many conserved miRNAs in the *Citrus* genome have more than one locus (Supplementary Fig. 3); such loci can be located on different chromosomes containing different precursor sequences, and mature miRNAs can arise from either the 5′ or 3′ arms of precursors (Fig. 1a). In addition, we identified a number of single-base variants of miRNAs in the four sRNA libraries. Such miRNAs exhibited moderate read counts; hence, they may not be derived from occasional sequencing errors, indicating that single nucleotide polymorphisms (SNP) of miRNAs might exist in plants, as reported previously^39^.

As small functional units, the biological functions of miRNAs may be altered by single nucleotide shifts in their precursor sequences, and in mature miRNA sequences in particular, driving the evolution of new miRNAs^40^. Elucidating the sequence variations of conserved miRNA isoforms could be quite helpful in the investigation of miRNA evolution in plants. Sequence alignment revealed that both SNPs and length differences of miRNAs (miR-LDs) could be observed (Fig. 1b; supplementary Fig. 4). In most miRNAs, the mature sequences of a given family exhibit a divergence of 1–3 nucleotides at both ends of the sequence. Seed sequences generally remain highly conservative, and SNPs are mainly located at mismatch positions. For example, the SNP sites observed in miR156/157 were identical or quite similar, causing diverse members of this miRNA family to target identical or similar genes, thus showing functional conservation (Fig. 1b,c). In contrast, the situation for the miR170/171 family was found to be complicated, with multi miR-LDs accompanied by divergent SNP sites potentially causing mismatches of miRNA-mRNA binding sites, resulting in changes in or loss of their target genes (supplementary Fig. 4) and even potential generation of new miRNAs^39^. These phenomena are consistent with previous reports on the miRNAs of both plants and animals^39^,^40^,^41^,^42^.

The remaining non-annotated sequences (538,928 reads; Supplementary Dataset S1) were assigned using MTide software (http://bis.zju.edu.cn/MTide/) for novel miRNA prediction. Finally, 312 candidate novel miRNAs were identified, and their characteristic hairpin structure formation ability was predicted. Considering that the availability of less stable anti-sense miRNA (miRNA*) sequence, indicating precise excision of a discrete miRNA/miRNA* duplex from the stem-loop precursor, is an important criterion for the validation of novel miRNAs^43^, we also searched for miRNAs* in our datasets; 261 miRNAs* (83.65%) for new *Citrus* miRNA candidates were found, providing evidence supporting them as new miRNAs. The remaining 51 predicted miRNAs without corresponding miRNAs* can be considered potential novel miRNAs based on the dominance of their read counts from one arm of the predicted stem-loops^43^ and the low detectability of corresponding miRNAs* for a large number of candidate miRNAs that exhibited only a few reads^39^,^44^. However, further verification approaches will be needed.

### Differential expression of miRNAs under B-sufficient and B-toxic conditions

**Figure 2:**
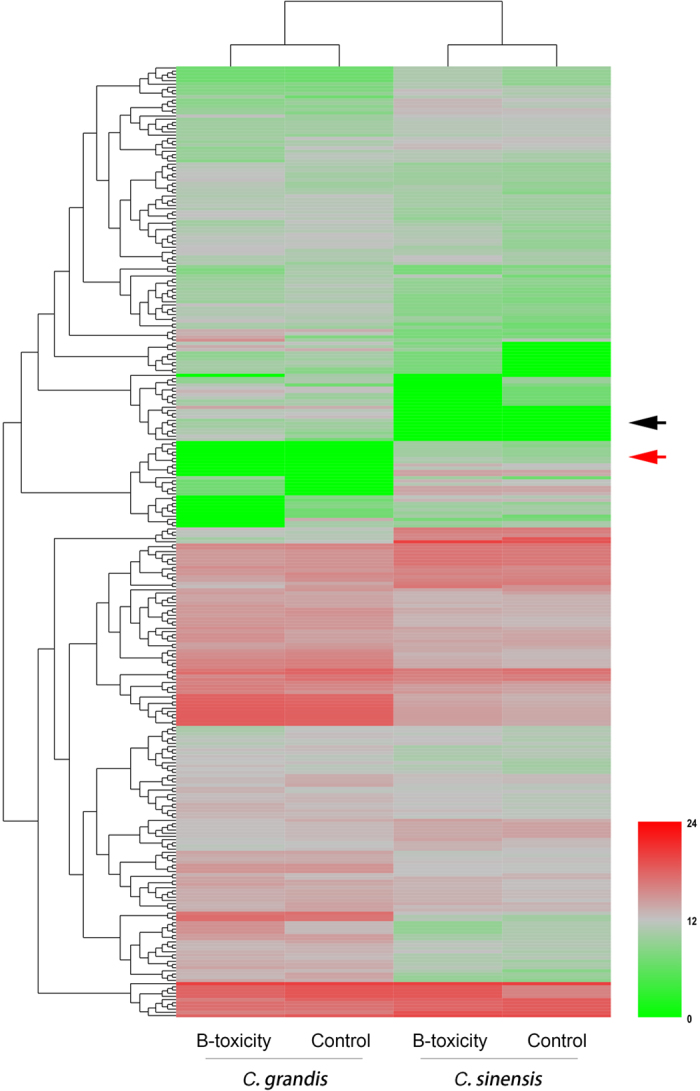
DEG clusters of differentially expressed miRNAs in *Citrus* leaves treated with different B levels. The red arrowhead showed miRNAs which specifically expressed in *C. sinensis*, whereas the black one indicated those in *C. grandis*.

IDEG6 was applied for the analysis of differentially expressed miRNAs^45^ because we used mixed samples for sRNA library construction and sequencing, without biological replicates. Our results showed species specificity of the miRNA expression patterns in the two *Citrus* species, even under different B levels (Fig. 2). However, our real interest was in miRNAs that were either shared by or specific to the two *Citrus* species, or were differentially expressed in response to different B treatments. We compared the miRNA expression levels in each species after treatment with different B levels and identified 51 (25 up- and 26 down-regulated) and 20 (16 up- and 4 down-regulated) differentially expressed miRNAs in *C. grandis* and *C. sinensis*, respectively, after B-toxic treatment. Among the differentially expressed miRNAs, one conserved miRNA (miR5156) expressed in *C. grandis* was species specific but was repressed under B-toxic conditions. Another novel miRNA (ci-miRN4) exhibited opposite expression patterns in the two species, whereas the rest were significantly up- or down-regulated only in either *C. grandis* or *C. sinensis* (Supplementary Dataset S2).

### Validation of miRNA expression patterns through Illumina sequencing

**Figure 3:**
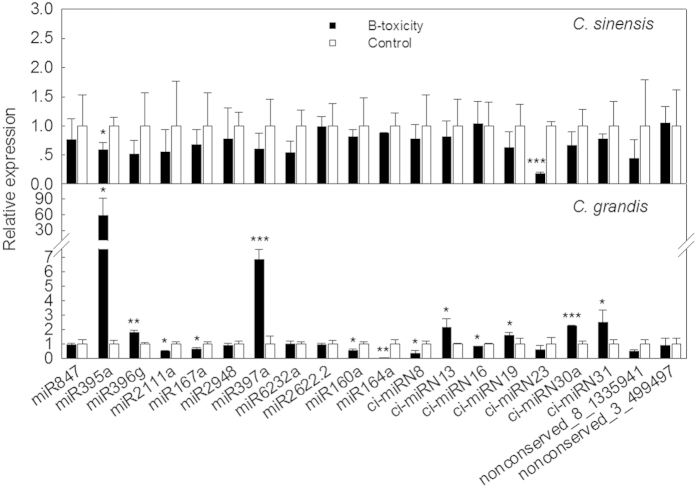
Relative abundance of miRNAs in *Citrus* leaves under B-sufficient and B-toxic conditions. MiRNA expression in B-sufficient and -toxic leaves was determined using stem-loop qRT-PCR. Results represent mean ± SD (n = 3). “*”, “**” and “***” indicate significant difference at *P* < 0.05, 0.01 and 0.001 level, respectively (the same below). All the values were expressed relative to the control.

To confirm the consistency of the deep sequencing results and comparative analyses, we verified the expression patterns of 20 miRNAs using stem-loop qRT-PCR. Among the differentially expressed miRNAs, miR395a and miR397a were the most significantly up-regulated in B-toxic *C. grandis* leaves, but both were down-regulated in B-toxic *C. sinensis* leaves (Fig. 3). Overall, the qRT-PCR results were similar to those from direct sequencing in 82.5% of cases. The exceptions were miR847, miR395a and miR160a in *C. sinensis* and miR6232a, miR2622.2, miR160a and a non-conserved ci-miRN16 in *C. grandis* (Fig. 3, Supplementary Dataset S2). Among these miRNAs, ci-miRN16 was down-regulated under B-toxicity when assayed via qRT-PCR, whereas it was up-regulated according to direct sequencing. This discrepancy may have resulted from the adaptor linkage efficiency in deep sequencing or PCR bias before sequencing^38^.

### Prediction of targets for differentially expressed miRNAs through GO analysis

Here, a total of 751 genes were predicted for 257 differentially expressed conserved and non-conserved miRNAs among the four libraries using bioinformatics. Because of the species and/or lineage specificity of miRNA expression in diverse plants and animals^46^,^47^,^48^,^49^,^50^,^51^, miRNAs that are specifically expressed in either *C. grandis* or *C. sinensis* and consistently expressed under B-sufficient and B-toxic conditions might be involved in the differences between the two *Citrus* species under B-toxic stress. However, the majority of such species-specific miRNAs are non-conservative and have no predicted targets. To better understand how miRNAs function in the response/adaptation of *Citrus* to long-term B-toxic stress, we therefore focused on target genes predicted from miRNAs that showed a discrepancy between B-sufficient and B-toxic conditions in each species. Finally, 102 (71 for *C. grandis* and 31 for *C. sinensis*, respectively) genes were obtained from the 71 (51 and 20 for *C. grandis* and *C. sinensis*, respectively) differentially expressed miRNAs identified in leaves (Supplementary Dataset S3). It is noteworthy that one miRNA might have several potential targets, which may either belong to the same family (e.g., genes targeted by miR397a) or have distinct predicted functions (e.g., genes targeted by miR847); conversely, a single gene can be targeted by several miRNAs, some of which are up-regulated, whereas others are down-regulated (e.g., *Ciclev10010074m* was predicted to be targeted by both miR6232a and ci-miRN16, which were down- and up-regulated, respectively, in *C. grandis*).

**Figure 4:**
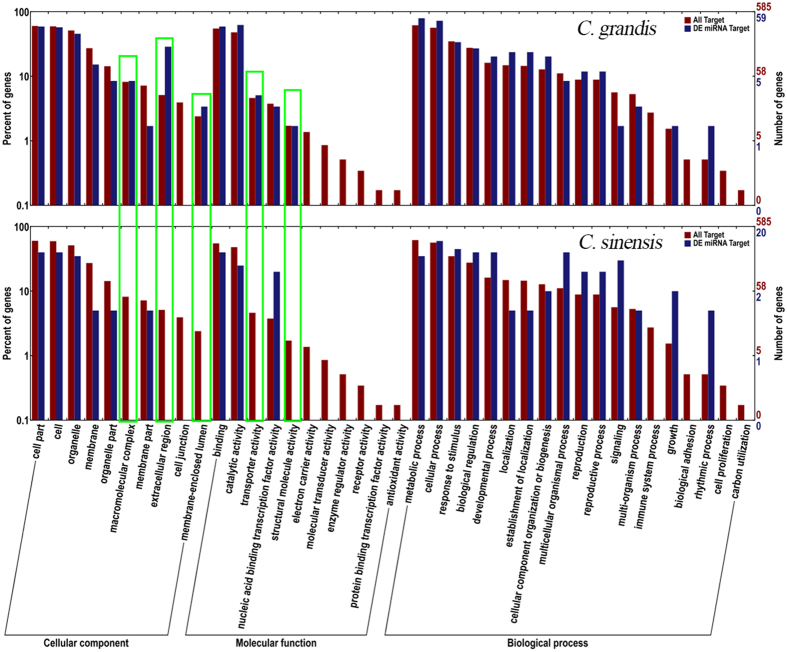
GO analysis of target genes predicted from differentially expressed miRNAs. Green rectangle indicated significant alternations of sample frequencies (Blue bars) of GO terms. Red bars displayed background frequencies of GO terms, in which the putative B-toxic-adaptation-relevant miRNA targets were enriched.

GO categories were then assigned to all the predicted targets according to the cellular component, molecular function and biological process categories (Fig. 4). The results indicated that noticeable changes in the sample frequencies of the GO terms, in which the potential targets of miRNAs that were differentially expressed under B-toxic stress were enriched, appeared between the two *Citrus* species in 5 categories. These categories were macromolecular complexes, extracellular regions and the membrane-enclosed lumen, which were classified as cellular components, and transport activity and structural molecule activity, which were classified as molecular functions.

### qRT-PCR relative expression analysis of target genes

**Figure 5:**
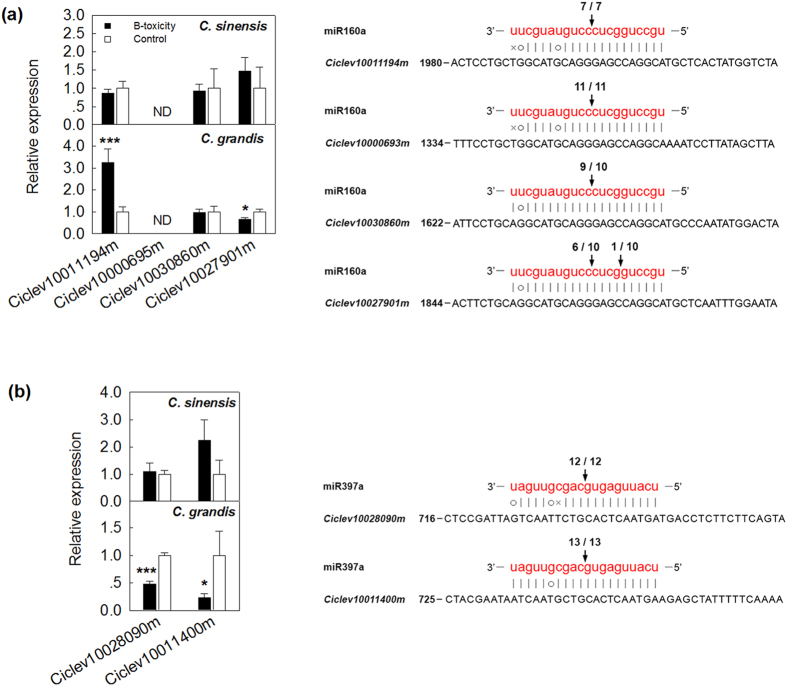
QRT-PCR expression profiles and experimental validation of the predicted mRNA targets for miR160a (a) and miR397a (b). The mRNA cleavage sites were determined by RLM-5′-RACE. The targeted mRNA sequences and mature miRNA sequences were shown. Vertical arrows indicated the 5′ termini of miRNA-mediated cleavage products, as identified by 5′-RACE, with the frequency of clones shown. *ND*, Not detected in controls.

Eighteen genes targeted by 11 differentially expressed miRNAs (seven conserved and four novel miRNAs) were assayed via qRT-PCR. Only six genes exhibited the expected changes in mRNA levels, suggesting that they might be regulated via miRNA-mediated cleavage under B-toxic stress. Two genes targeted by miR164a and one targeted by miR160a were not detected in control leaves, despite being detected at extremely low levels relative to *actin* in B-toxic leaves. The expression of three other target genes was positively correlated with the levels of their corresponding miRNAs. The remaining six target genes maintained a relatively stable expression level (Table 2, Fig. 5a,b). Overall, our results are consistent with previous reports in *Citrus*^36^ and in *Arabidopsis*^52^, validating the low efficiency of expression profiles for predicting miRNA target genes.

### Verification of potential ci-miRNA target genes with RLM-RACE

To investigate the confidence of the predicted targets of miRNAs, we experimentally verified the cleavage of selected targets through modified 5′-RACE analysis. The RLM-5′-RACE procedure was successfully used to map the cleavage sites in six predicted miRNA target genes. *Ciclev10011194m*, *Ciclev10030860m*, *Ciclev10000695m* and *Ciclev10027901m* were confirmed as real targets of miR160a, and *Ciclev10038090m* and *Ciclev10011400m* were confirmed as real targets of miR397a because all the 5′-ends of the mRNA fragments mapped to the nucleotide that paired with the tenth nucleotide of each miRNA with a higher frequency than was observed for each pairing oligo (Fig. 5). *Ciclev10011194m* and *Ciclev10030860m* are homologous to the *Oryza sativa* auxin response factor (ARF) gene *ARF18*, whereas *Ciclev10000695m* and *Ciclev10027901m* are similar to *AthARF17*and *AthARF10*, respectively. *Ciclev10038090m* and *Ciclev10011400m* are similar to isoforms of *Laccase-17* (*LAC17*) and *LAC4*, respectively.

### The expression of miR397a affects cell-wall polysaccharide deposition in *Citrus* leaf veins

**Figure 6:**
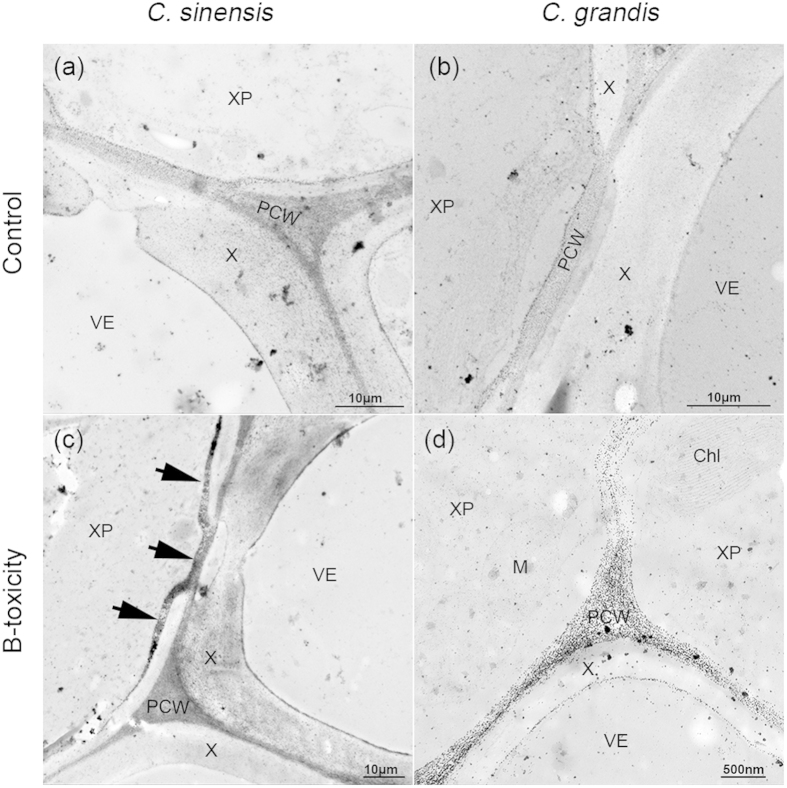
Crossed sections of the leaf mid-veins stained using PASH produce. B-toxic treatment led to the deposition of secondary cell-wall components in the xylem parenchyma cells (XP) near the pits of vessel elements (VE) in *C. sinensis* (**c**), *arrowheads*); but restrained the development of VEs in *C. grandis* (**d**). Chl, chloroplast; M, mitochondria; PCW, primary cell walls.

Given that both *LAC4* and *LAC17* contribute to secondary cell-wall synthesis in *Arabidopsis* stems and that *LAC17* is involved in the deposition of G lignin units in fibres^53^, we examined the cell-wall polysaccharides of vascular bundles in the two *Citrus* species treated with different B levels. In B-tolerant *C. sinensis*, the xylogens of vessel elements under B-toxic conditions were similar to those under B-sufficient conditions. Moreover, secondary deposition of cell-wall polysaccharides could often be observed in regions near the pits of vessel elements (Fig. 6a,c). Intriguingly, the components of the deposited secondary cell walls were so sensitive to the silver reagent that they appeared to be extremely condensed under transmission electron microscopy (Fig. 6c), suggesting a significant difference in their polysaccharide structure or biochemical components from those of xylogens. However, B-toxic treatment resulted in poorly developed vessel elements in *C. grandis*, with fewer silver particles deposited in the secondary cell wall (Fig. 6b,d).

## Discussion

As important post-transcriptional regulators, miRNAs have been extensively studied in the past several years. A great number of studies have demonstrated that miRNAs are involved in the adaptive responses of plants to various biotic and abiotic stresses^54^,^55^,^56^,^57^,^58^. However, only a few miRNAs that respond to B-toxic stress have been identified in barley^35^ thus far, and there are no reports of B-toxicity-responsive miRNAs in woody plant species. Systematic evaluation of miRNAs in *Citrus* under B-toxicity will provide insights into the mechanisms underlying the tolerance to and/or the molecular basis of B-toxicity in woody plants.

Here, through Solexa deep sequencing, a total of 699 (387 conserved and 312 novel) candidate miRNAs were identified from *Citrus* leaves treated with different B levels. Among the identified miRNAs, 51 (23 conserved and 28 novel) were differentially expressed in the B-toxicity-treated leaves of intolerant *C. grandis* and 20 (6 conserved and 14 novel) in those of tolerant *C. sinensis* (Supplementary Dataset S2), demonstrating that miRNAs play an important role in *Citrus* responses to B-toxicity.

### Sequence variations of miRNAs in *Citrus*

Variation in the seed sequences of miRNAs may be an important factor driving the evolution of miRNAs in plants^39^. As reported for *Arabidopsis*^40^,^41^,^42^, both miR-SNPs and miR-LDs appeared in identified miRNAs that were either conserved or non-conserved in *Citrus* (Supplementary Dataset S1). In most cases, the members of a miRNA family are located in clusters on different chromosomes, with identical SNP sites mostly being located beyond the seed sequences (i.e., miR156/157, Fig. 1b). However, some miRNA family members were found to show divergence in their locations and sequences (i.e., miR170/171, Supplementary Fig. 4), possibly due to the high heterozygosity of the examined species, which might have arisen as a result of long-term selection via cross-breeding in *Citrus*. Interestingly, some of the miRNAs (i.e., miR156c and miR171h) that exhibited extensive variations in sequences beyond the seed region (Fig. 1b; Supplementary Fig. 4) were expressed in a species-specific manner at extremely low levels (Supplementary Dataset S1) and presented no predicted targets, thus allowing plants to accumulate mutations under selective pressure for miRNA evolution, indicating that species specificity might be involved in miRNA evolution.

### Interactions between miRNAs and their predicted targets

At present, the most efficient way to assess and define the putative functions of a given miRNA in plants is target prediction using bioinformatics, which is based on the high degree of homology between miRNAs and their target sequences^59^. Using computational techniques, a total of 102 genes were predicted from 26 B-toxicity-responsive miRNAs in *Citrus* (Supplementary Dataset S3). As noted by Meyers *et al.*^43^, it is very difficult to validate the true targets of a given miRNA, and the outcomes of both our pRT-PCR and 5′-RACE assays were disappointing: only 6 of 18 (33.33%) selected target genes exhibited the expected changes in mRNA levels when examined via qRT-PCR (Table 2; Fig. 5a,b; Supplementary Dataset S1), and only 6 of 29 (20.69%) genes were confirmed as true targets cleaved via miRNA-mediated cleavage when RLM-5′-RACE analysis was applied.

The contradictions in our results might arise from the diversity of miRNA regulatory mechanisms as well as the complexity of the gene regulation network at a genomic scale. It has been known for quite some time that plant miRNAs regulate their specific target mRNAs via cleavage based on their near-perfect complementarity to their target genes^26^. However, it was not until very recently that Li *et al.*^28^ experimentally demonstrated translation inhibition of targets by miRNAs in *Arabidopsis*. These two theories of miRNA regulation, together, might explain most of our failures to validate miRNA targets using either the qRT-PCR or 5′-RACE approach.

It is interesting that some miRNA targets, such as *Ciclev10027901*, that were confirmed as real targets of miRNAs according to the 5′-RACE approach and were therefore expected to be negatively regulated at the transcription level were instead found to be positively expressed as their miRNA levels were increased according to qRT-PCR (Figs 3 and 5a). Considering that a single gene can be targeted by several miRNAs simultaneously and that another potential miR160a cleavage site, located approx. 60 bp downstream from the verified exact cleavage site, was identified in our 5′-RACE experiments, this contradiction might be attributed to the complex gene regulation network.

Nevertheless, our data and those of others^37^,^38^,^39^,^60^ support the prevailing model indicating that miRNAs regulate specific targets through cleavage in plants.

### MiRNAs associated with the response to B-toxicity

High-throughput sequencing technology has recently been successfully applied to identify miRNAs on a genomic scale in plants under either B-toxic (in barley leaves and roots)^35^ or B-deficient (in *Citrus* roots)^36^ stress. Unfortunately, the published expression profiles of miRNAs responsive to B-stress have little in common with ours. This difference might be due to the species specificity and spatio-temporal specificity of miRNA expression in plants. It could also be caused by different expression patterns of miRNAs in the leaves of barley and *Citrus* under B-toxicity, which might indicate different tolerance mechanisms between herbaceous and woody plants.

Among these differentially expressed miRNAs, miR395a and miR397a were confirmed by qRT-PCR to be the most significantly up-regulated and exhibited negative expression patterns between *C. grandis* and *C. sinensis* (Fig. 3) when exposed to B-toxicity. In *Arabidopsis*, miR395 targets genes belonging to the ATP sulfurylase gene family^61^ as well as sulfate transporters^55^ to modulates sulfate accumulation and allocation^34^. In *Citrus*, miR395a was predicted to target *Ciclev10004931m*, described as 3-ketoacyl-CoA thiolase 2, which is presumed to respond to wounding or positively regulate the abscisic acid-activated signalling pathway. However, its predicted cleavage site was confirmed to be false via the 5′-RACE approach. Considering its high expression level and the fact that B-toxicity resulted in leaf senescence in *C. grandis*, miR395a might regulate its target *Ciclev10004931m* via translation inhibition.

Using 5′-RACE, we confirmed two genes (*LAC4* and *LAC17*) as real targets of miR397a. In *Arabidopsis*, *in vivo* expression analysis indicated that both miR397 and its targets (*LAC4* and *LAC17*) are mainly expressed in vascular tissues^53^,^62^; over-expression of miR397b causes a reduction of the lignification of vascular and interfascicular tissues^62^. Similarly, an increase in the expression of miR397a was accompanied by down-regulation of both *LAC4* and *LAC17* after B-toxicity treatment, leading to poor development of vessel elements in the vascular bundles of *C. grandis* (Fig. 6d), suggesting that both miR397a and its two targets might also be specifically expressed in vascular bundles in *Citrus*. Given that *LAC4* and *LAC17* cluster in different groups based on multiple amino acid sequence alignment^63^,^64^, they might have different biological functions. In a study by Wang *et al.*^62^, *LAC4* was found to be involved in lignin biosynthesis and the seed yield, whereas loss-of function single mutants of *LAC17* displayed no significant morphological changes. In the current study, *LAC17* remained at the same level, whereas *LAC4* was up-regulated approx. 2-fold in *C. sinensis* when treated with toxic levels of B. A PASH procedure performed on B-toxicity-treated leaf veins of *C. sinensis* revealed that secondary cell-wall polysaccharides in xylem parenchyma cells were deposited in regions near the pits of vessel elements but reacted differently to the silver reagent than did xylogen (Fig. 6a,c), suggesting that *LAC4* might have an additional function in secondary cell-wall synthesis. Thus, the modification of cell-wall structures and components in the xylem resulting from the modulation of both *LAC4* and *LAC17* by miR397a might play an important role in the *Citrus* response to long-term B-toxicity

Both miR160a and miR164a were significantly down-regulated in B-toxic *C. grandis* leaves (Fig. 3). Using bioinformatics, 6 ESTs homologous to the *ARF* gene family were predicted to be targeted by miR160a (Supplementary Dataset S3), among which Ciclev10000693m, Ciclev10000695m and Ciclev10000696m might be copies of the same gene with different chromosomal loci based on DNA sequence alignment and ORF prediction. Therefore, only *Ciclev10000695m* and the other 3 potential target genes were included in the subsequently experiments, and all were confirmed as real targets of miR160a. Based on the extensive documentation of the involvement of *ARFs* in auxin signalling^65^,^66^, miR160a might play a role in auxin signalling in the *Citrus* response to B-toxicity; however, it is difficult to draw a conclusion because of the opposite expression patterns of the four *ARF* genes (Fig. 5a). For miR164a, two genes, encoding a NAC domain protein (*Ciclev10008619m*) and polygalacturonase (*Ciclev10033386m*), were predicted as targets. In plants, polygalacturonase is responsible for pectin solubilization and depolymerization^67^. Down-regulation of miR164a in response to B-toxicity might increase polygalacturonase activity, resulting in cell-wall modifications. This notion was supported by a recent report by Ghanati and Heidarabadi^6^, who found that an increased B supply led to a significant decrease in the cell or cell-wall dry weight and a reduction of the relative amounts of major wall components, including pectin, cellulose and hemicellulose B.

Most of the other differentially expressed miRNAs identified in the present study were predicted to target diverse genes related to transcription factors that remain to be determined and will be further discussed elsewhere.

### Possible tolerance mechanisms of *Citrus* to long-term B-toxicity

The most widely accepted theory of B transport from the roots to the shoots of plants involves passive transport via mass flow within the transpiration stream^68^. Upon reaching the leaf, free B in the transpiration stream will first flow into the phloem, before being transported into mesophyll tissue, either passively or actively, in dicotyledons. Therefore, we have reason to believe that B may first accumulate in the phloem, generating toxic effects, which has been confirmed by the finding that B-toxic treatment results in limited toxic effects in leaf veins through triggering programmed cell death of phloem tissue in intolerant *C. grandis*^23^.

In the present study, B-toxic treatment resulted in secondary deposition of cell-wall components in regions near the pits of vessel elements in tolerant *C. sinensis* and poorly developed vessel elements in intolerant *C. grandis* (Fig. 6), indicating that modifications of cell-wall structures and components in the xylem might be involved in mitigating B-toxicity to the phloem.

It has been demonstrated that in plants, B forms the most stable diesters with *cis*-diols on furanoid rings^69^, participating in cell-wall assembly by cross-linking polysaccharide monomers into dimers^70^. Given that B exists as boric acid under physiological conditions and can form esters and complexes with a wide variety of hydroxyl-rich compounds^71^, cell-wall polysaccharides as well as other cell-wall components, such as hydroxyl-proline-rich glycoproteins^4^, could be considered candidates for B binding, even with weak bonds. Therefore, alterations of cell-wall structures and components in the xylem might restrict the inflow of free B from the xylem into the phloem in tolerant *C. sinensis*. Indeed, the expected lower level of free B in B-toxicity-treated leaves of *C. sinensis* has been detected^23^.

Above all, the results reported herein illustrate that through the modulation of *LAC17* and *LAC4*, miR397a plays a pivotal role in secondary cell-wall biosynthesis in vascular bundles, thereby contributing to the adaptation of woody *Citrus* to long-term B-toxicity.

## Materials and Methods

### Plant culture and treatments

Plant culture and B treatments were performed as previously reported^23^. Briefly, 11-week-old seedlings were sand cultured with a nutrient solution containing 10 (control) or 400 (B-toxic) μM H_3_BO_3_ every other day for 15 weeks. Fully expanded leaves excised at 1/3 the height of the plants were immediately frozen in liquid N_2_ at the end of the culture experiment and stored at −80 °C until RNA extraction. Mid-vein samples were directly fixed with 2.5% glutaraldehyde in 0.1 M phosphate buffer (PBS, pH 7.4) at 4 °C for 24 h for anatomical analyses.

### RNA isolation and quality assessment

Total RNA was extracted using the TRIzol reagent (Invitrogen, Carlsbad, CA) according to the manufacturer’s instructions, with minor modifications (3 M sodium acetate was used for the removal of polysaccharides). The quantity and purity of the total RNA were analysed using a NanoDrop 2000 spectrophotometer (Thermo scientific, USA) and an Agilent 2100 bioanalyser (USA) with the RNA Integrity Number (RIN) > 8.0. The total RNA was divided, stored at −80 °C, and used for high-throughput sequencing, qRT-PCR and RACE amplification.

### Small RNA library construction and high-throughput sequencing

Approximately 1 μg of mixed total RNA from five replicates was used to prepare a small RNA (sRNA) library with the TruSeq Small RNA Sample Prep Kit (Illumina, USA) according to the manufacturer’s protocol. The sRNA libraries were gel-purified in 6% PAGE gels, and their concentration was then diluted to 2 ng/μl. The insert size of the sRNA libraries was tested with an Agilent 2100 bioanalyser. After accurate quantification via qRT-PCR, single-end sequencing was performed on an Illumina HiSeq 2500.

### Data processing, sRNA annotation and miRNA identification

The raw reads obtained from Illumina sequencing were subjected to the Illumina pipeline filter, and the datasets were further processed to remove adapter dimers, junk and low-complexity sequences, common RNA families (rRNA, tRNA, snRNA, snoRNA) and repeats, as reported previously^72^. The remaining sequences were subjected to BLAST searches against miRBase 21 (http://www.mirbase.org/) to identify known miRNAs. Non-annotated sequences of 18–30 nucleotides (nt) in length were mapped to the *Citrus clementina* genome (JGI version 1.0, http://www.phytozome.org/clementine.php) with the MTide program (http://bis.zju.edu.cn/MTide) to identify novel sequences with stem-loop precursors, as described by Zhang *et al.*^73^. MiRNA prediction was performed according the key criteria previously used by Meyers *et al.*^43^.

### Differential expression analysis of miRNAs

For the analysis of miRNA expression, both the fold-change and *P*-value of each identified miRNA in the control and B-toxic libraries were calculated as previously reported by Lu *et al.*^36^. The *P*-value was then adjusted to the false discovery rate (FDR) using the Benjamini Hochberg Method. A 2-fold cut-off was set to determine up- and down-regulated miRNAs, in addition to a FDR of less than 0.01.

### Prediction of miRNA target genes

The prediction of genes targeted by differentially expressed miRNAs was performed with TargetFinder based on rules suggested by Allen *et al.*^61^ and Schwab *et al.*^74^.

### GO analysis of miRNA targets

All predicted targets were mapped to GO terms in the GO database (http://www.geneontology.org/), and gene numbers were calculated for each term. The GO results were expressed according to three categories: cellular component, molecular function and biological process^75^.

### MiRNA assay using stem-loop qRT-PCR

To monitor the relative abundance of miRNAs obtained from Illumina sequencing, 20 miRNAs (11 conservative and 9 non-conservative) were selected to perform qRT-PCR using *actin* (AEK97331.1) as an internal control. Stem-loop RT primers were designed according to Xu *et al.*^76^. MiRNA-specific primers (forward) were designed according Chen *et al.*^77^, and their quality was assessed using Primer Software (PREMIER Biosoft International, USA, Version 5.0). All the primers employed for qRT-PCR are listed in Supplementary Table 1.

For stem-loop qRT-PCR, approx. 1 μg of total RNA was used to create a reverse transcription pool with the TaqMan® MicroRNA Assay Kit (Takara, Japan) according to the manufacturer’s instructions. qRT-PCR was then performed as previously described by Lu *et al.*^36^, using the miRNA-specific forward primer and a Universal Reverse Primer (Supplementary Table 1). The cycling conditions were as follows: 10 min at 95 °C, followed by 40 cycles of 95 °C for 15 s and 60 °C for 60 s. The samples subjected to qRT-PCR were run in three biological replicates with two technical replicates. Relative miRNA expression was calculated using the ddCt algorithm. *Actin* was employed as an internal standard for the normalization of miRNA expression, and leaves from control plants were used as a reference sample (set as 1). As total RNA was used for reverse transcription, melting curve analysis followed by agarose gel electrophoresis was conducted to determine the purity and fragment size of the amplicons.

### Detection of target mRNAs via qRT-PCR

qRT-PCR analysis of target gene expression was performed as previously described^78^ with the Mastercycler Ep Realplex System (Eppendorf, Germany). Gene-specific forward and reverse primers were designed approx. 200 bp up- and down-stream from potential cleavage sites, to produce an expected fragment of approx. 100–300 bp in size. The quality of all primer pairs was assessed using Primer Software and the primers are provided in Supplementary Table 2.

### Verification of miRNA cleavage sites via 5′-RACE

To verify the nature of potential miRNA targets and to examine how the miRNAs regulate their target genes, an RNA ligase-mediated 5′ rapid amplification of cDNA ends (RLM-5′-RACE) experiment was set up. RLM-5′-RACE was carried out following the instructions of the GeneRacer Kit (Invitrogen), as described by Yin *et al.*^38^. Briefly, poly(A)^+^ mRNA was isolated from total RNA using the polyAtract mRNA isolation system III (Promega, Madison, WI). The GeneRacer RNA Oligo adapter was directly ligated to poly(A)^+^ mRNA without calf intestinal phosphatase and tobacco acid pyrophosphatase treatment, according to the manufacturer’s instructions. The GeneRacer OligodT primer was then used to synthesize RACE-ready first-strand cDNA in a reverse transcription reaction. First-round PCR was performed using the GeneRacer 5′ primer and a gene-specific primer, followed by second-round PCR with the GeneRacer 5′ Nested Primer and a gene-specific nested primer, which was designed using Primer 5.0 software and was located 300–800 bp downstream from the potential cleavage sites. The gene-specific and nested primers used in these assays are listed in Supplementary Table 3. The nested PCR products were gel-purified, T-cloned and sequenced.

### Cytochemical localization of cell-wall polysaccharides and electron microscopy

For anatomical analyses, mid-vein samples were post-fixed with 1% osmium tetroxide for 1.5 h at 4 °C. Tissue blocks were rinsed with 0.1 M PBS (pH 7.4), then dehydrated in a graded ethanol series, and the ethanol was subsequently replaced through four changes of acetone. The samples were embedded in an Epon resin mixture. Sections of 75 nm were obtained with a Leica EM UC6 ultra-microtome (Germany) and transferred to fomvar-carbon-coated nickel grids, then stained using a modified Pickett-Heaps procedure (PASH staining) as described by VanDerWoude *et al.*^79^. The prepared specimens were examined at 80 kV with a Hitachi 7700 electron microscope (Japan).

### Experimental design and statistical analysis

A completely randomized design was applied with 20 pot seedlings per B-treatment. The experiments were performed with 3–5 replicates. Differences between treatments were subjected to independent t-tests using IBM SPSS statistics software (Version 22).

## Additional Information

**Accession codes:** The sRNA sequence data from this study have been submitted to Gene Expression Omnibus (GEO) under accession GSE74434 at http://www.ncbi.nlm.nih.gov/geo/query/acc.cgi?acc=GSE74434.

**How to cite this article**: Huang, J.-H. *et al.* Illumina microRNA profiles reveal the involvement of miR397a in *Citrus* adaptation to long-term boron toxicity via modulating secondary cell-wall biosynthesis. *Sci. Rep.* **6**, 22900; doi: 10.1038/srep22900 (2016).

## Supplementary Material

Supplementary Information

Dataset S1

Dataset S2

Dataset S3

## Figures and Tables

**Table 1:** Summary of sRNAs from the leaf samples of *C. sinensis* and *C. grandis* treated with sufficient- and toxic-B level.

		C. sinensis		C. grandis	
		Control	B-toxicity	Control	B-toxicity
Raw reads		19886678	13833254	20680130	16889149
Clean reads	Total	11188651	8185253	11438525	8350155
	Genome	1979020(17.69%)	1562702(19.09%)	2091835(18.29%)	1500284(17.97%)
	rRNA	6587115(58.87%)	4643219(56.73%)	5944199(51.97%)	4439710(53.17%)
	scRNA	0(0.00%)	0(0.00%)	0(0.00%)	0(0.00%)
	snRNA	3011(0.03%)	2342(0.03%)	2062(0.02%)	1638(0.02%)
	snoRNA	659(0.01%)	752(0.01%)	795(0.01%)	982(0.01%)
	tRNA	252937(2.26%)	224480(2.74%)	277303(2.22%)	202708(2.43%)
	Rep.-base	22168(0.20%)	18196(0.22%)	24786(0.22%)	21681(0.26%)
	Non-annotated	2343741(20.95%)	1733562(21.18%)	3097545(27.08%)	2183152(26.15%)
Unique number		1593866	1214103	1988267	1350479

**Table 2:** Relative expression of miRNA targets under B-toxicity in *Citrus* using qRT-PCR.

miRNAs	Target gene	Description	Expression level (Mean ± SD)	
			*C. sinensis*	*C. grandis*
miR164a	Ciclev10008619m	NAC domain protein	ND	ND
	Ciclev10033386m	Polygalacturonase	ND	ND
miR395a	Ciclev10004931m	3-ketoacyl-CoA thiolase 2	0.640 ± 0.044	1.381 ± 0.181^*^
miR2111a	Ciclev10015288m	F-box/kelch-repeat protein	0.761 ± 0.241	5.290 ± 0.789^***^
miR396g	Ciclev10017846m	Ubiquitin-protein ligase activity	0.566 ± 0.202^*^	0.815 ± 0.697
Ci-miRN16	Ciclev10010074m	Cytochrome c oxidase subunit	0.828 ± 0.065	2.762 ± 0.406^**^
	Ciclev10010096m	Cytochrome c oxidase subunit	0.455 ± 0.134	1.239 ± 0.510
Ci-miRN8	Ciclev10032738m	Ubiquitin receptor	0.880 ± 0.219	1.948 ± 0.602
Ci-miRN31	Ciclev10012377m	Transcription factor	1.540 ± 0.429	0.812 ± 0.325
	Ciclev10027736m	DNA/RNA helicase protein isoform 1	1.581 ± 0.916	2.634 ± 0.550^*^
Ci-miRN2622.2	Ciclev10021082m	DNA/RNA helicase protein isoform 1	0.978 ± 0.144	0.975 ± 0.250
Ci-miRN23	Ciclev10009779m	Blue copper protein	0.801 ± 0.113	0.960 ± 0.331

Note: Significance difference was tested between control and B-toxic treatment using independent sample *t*-test (n = 3). “*”, “**” and “***” indicated significant difference at *P* < 0.05, 0.01 and 0.001 level, respectively. *ND*, NOT detected in controls.
