# Supplementary material for: Illumina microRNA profiles reveal the involvement of miR397a in Citrus adaptation to long-term boron toxicity via modulating secondary cell-wall biosynthesis
Source: Sci Rep. 2016 Mar 10;6:22900. doi: 10.1038/srep22900 (PMC4790630; doi:10.1038/srep22900)
Supplement: Supplementary Information [file srep22900-s1.pdf]

# Illumina microRNA profiles revealed involvement of miR397a in *Citrus* adaptation to long-term boron-toxicity via modulating secondary cell wall biosynthesis

Jing-hao Huang, Yi-ping Qi, Shou-xing Wen, Peng Guo, Xiao-min Chen, Li-song Chen*

* Corresponding author (E-mail: lisongchen2002@hotmail.com)

## Supplementary Figures and Tables

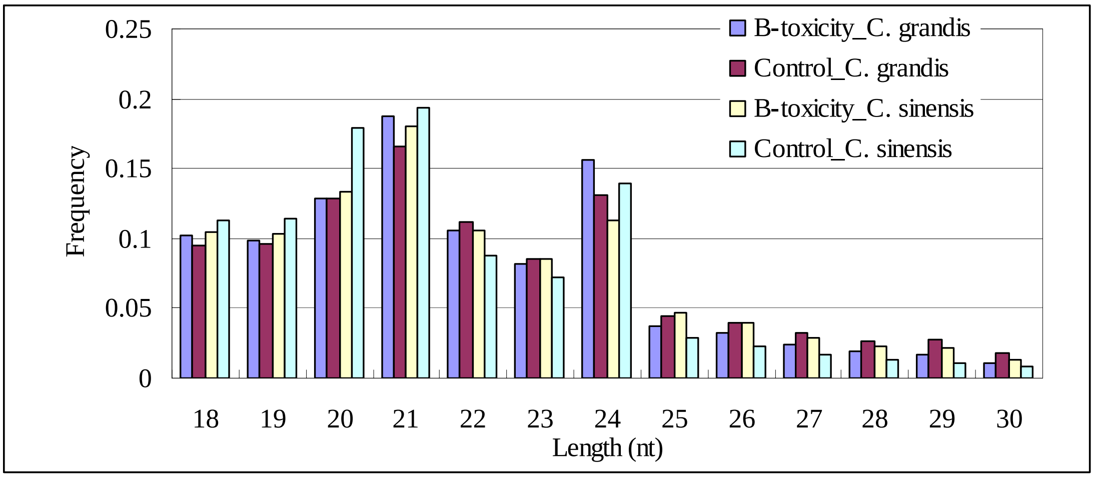


**Fig. 1 Size distribution of sRNAs of *C. grandis* and *C. sinensis* treated with different B level**.

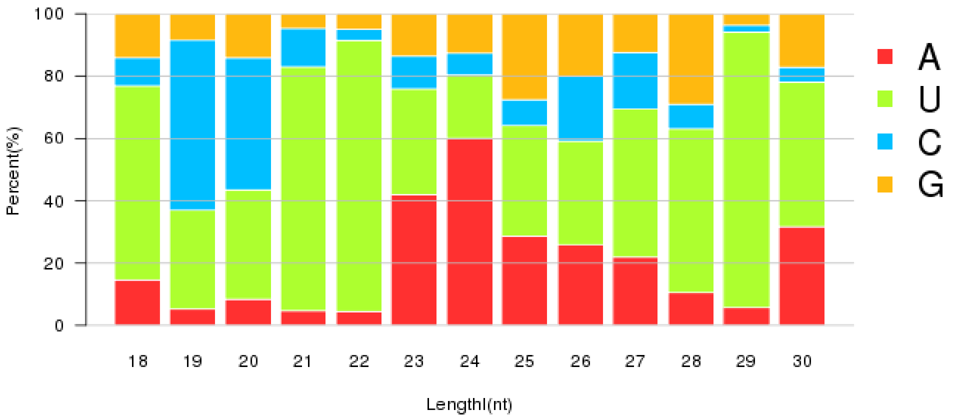


**Fig. 2 First nucleotide bias of miRNAs with different length**.

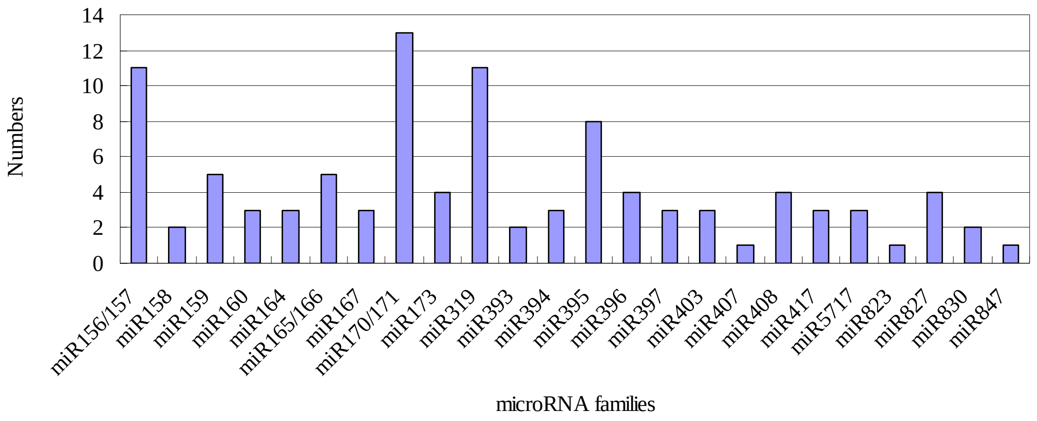


**Fig. 3 Numbers of identical miRNA member in each family in *Citrus* plants**

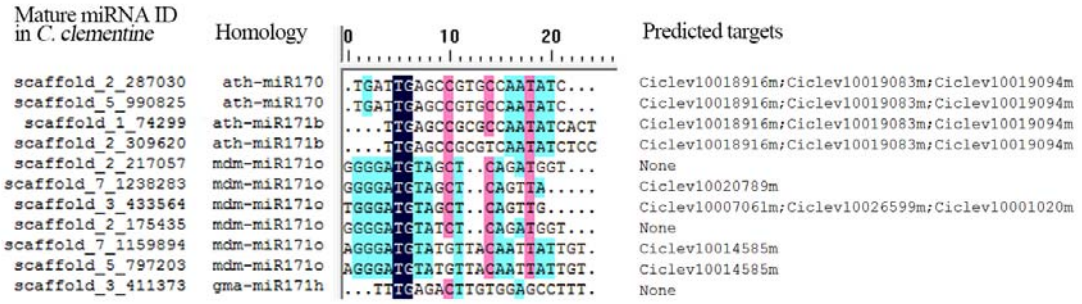


**Fig. 4 Alignment of miR170/171 members identified from *C. clementine* geneome using DNAMAN software (version 7)**. Divergent SNP sites with length variations in mature miRNA sequences could result in change or loss of their targets.

**Supplementary Table 1 RT primers and specify primers for miRNAs stem-loop qRT-PCR**

| | Specific forward primer (5'-3') | RT primer sequence (5'-3') |
|---|---|---|
| miR160a | GATTGCCTGGCTCCCTGTATG | GTCGTATCCAGTGCAGGGTCCGAGGTATTCGCACTGGATACGACAAGCAT |
| miR164a | GAATCTGGAGAAGCAGGGCA | GTCGTATCCAGTGCAGGGTCCGAGGTATTCGCACTGGATACGACTGCACG |
| miR167a | ACTTGAAGCTGCCAGCATGATCT | GTCGTATCCAGTGCAGGGTCCGAGGTATTCGCACTGGATACGACTAAGAT |
| miR2111a | CTCGTAATCTGCATCCTGAGGT | GTCGTATCCAGTGCAGGGTCCGAGGTATTCGCACTGGATACGACCAAACC |
| miR2622.2 | CGATTTGTGTGGTTGTTGTTGA | GTCGTATCCAGTGCAGGGTCCGAGGTATTCGCACTGGATACGACACTCAA |
| miR2948 | CTAGTGGGAGTGAGGAGTGTGGA | GTCGTATCCAGTGCAGGGTCCGAGGTATTCGCACTGGATACGACAATCCA |
| miR395a | TATGCTGAAGTGTTTGGGGGAA | GTCGTATCCAGTGCAGGGTCCGAGGTATTCGCACTGGATACGACGAGTTC |
| miR396g | GCCTGTTCAAGAAAGCTGTGGA | GTCGTATCCAGTGCAGGGTCCGAGGTATTCGCACTGGATACGACTTTTCC |
| miR397b | GGTCTTATTGAGTGCAGCGTTG | GTCGTATCCAGTGCAGGGTCCGAGGTATTCGCACTGGATACGACATCAAC |
| miR6232a | CACGTGGATGTAACTGTGGCAC | GTCGTATCCAGTGCAGGGTCCGAGGTATTCGCACTGGATACGACACCGTG |
| miR847 | GACGACTTGATGATGCTGCA | GTCGTATCCAGTGCAGGGTCCGAGGTATTCGCACTGGATACGACAATGCA |
| ci-miRN13 | GCCCGCAGTTTTAAAATTTTCTC | GTCGTATCCAGTGCAGGGTCCGAGGTATTCGCACTGGATACGACTCTGAG |
| ci-miRN16 | TGACTGGATGCAACTGTGGTAC | GTCGTATCCAGTGCAGGGTCCGAGGTATTCGCACTGGATACGACCCGTAC |
| ci-miRN19 | ACTACCGTGCCACAGTTGTATCC | GTCGTATCCAGTGCAGGGTCCGAGGTATTCGCACTGGATACGACGCTGGA |
| ci-miRN23 | GTCTCGAATGGATAATGAACG | GTCGTATCCAGTGCAGGGTCCGAGGTATTCGCACTGGATACGACACACGT |
| ci-miRN30a | GCGTCTTGTTGAGTGTGTATGTT | GTCGTATCCAGTGCAGGGTCCGAGGTATTCGCACTGGATACGACTGTAAC |
| ci-miRN31 | CGATTTCTTCATGAGAGCTGGC | GTCGTATCCAGTGCAGGGTCCGAGGTATTCGCACTGGATACGACTGGCCA |
| ci-miRN8 | CGGAGGAATTGTGTATTTTTGAA | GTCGTATCCAGTGCAGGGTCCGAGGTATTCGCACTGGATACGACCTTTTTC |
| nonconserved_3_499497 | TCCGGTTCTTTTTGCTACTTCTAC | GTCGTATCCAGTGCAGGGTCCGAGGTATTCGCACTGGATACGACCAGTAG |
| nonconserved_8_1335941 | GCCGCTAGAGAAAGATGAGAG | GTCGTATCCAGTGCAGGGTCCGAGGTATTCGCACTGGATACGACGTCTCT |
| Universal Reverse Primer | GTGCAGGGTCCGAGGT | |
| β-actin | AGAACTATGAACTGCCTGATGGC | GCTTGGAGCAAGTGCTGTGATT |

**Supplementary Table 2  Specific primers for qRT-PCR （5'-3'）**

| | |
|---|---|
| β-actin Forward | AGAACTATGAACTGCCTGATGGC |
| β-actin Reverse | GCTTGGAGCAAGTGCTGTGATT |
| Ciclev10011194m Forward | GAAGTCAAGACTGCCACAACCA |
| Ciclev10011194m Reverse | TTGTGGGTTTAGCAGCACGATC |
| Ciclev10000695m Forward | TCTTTCCAGTGAACGGTTTAGG |
| Ciclev10000695m Reverse | GACATCTGATGATTATTCTCGCTTA |
| Ciclev10030860m Forward | TAACCAACTTCCAACACCATCAT |
| Ciclev10030860m Reverse | TGAAAGCCCAGTGGAAACAG |
| Ciclev10027901m Forward | TCACCAGCAAGAAAGAAGTT |
| Ciclev10027901m Reverse | CTGGATAGAAACAGCCCTGA |
| Ciclev10004931m Forward | GTGACAAGGAAGGAGCAAGACC |
| Ciclev10004931m Reverse | CAACTTCGCCAACTCTGATACTG |
| Ciclev10017846m Forward | CGTGCCGTCTGTAAAAGATGGTATG |
| Ciclev10017846m Reverse | TTTGATGAGGGGTGTTTTTTGGTTG |
| Ciclev10028090m Forward | CTTCTCCGATTAGTCAATTCTGC |
| Ciclev10028090m Reverse | ATGACTAGCGTTTCGGTCTCAA |
| Ciclev10011400m Forward | CGTCACAGGGAGGCTTTACATT |
| Ciclev10011400m Reverse | TGGTTTAACATAAGTGGCATCA |
| Ciclev10015288m Forward | AAGGCAGGCGTGGTGGTT |
| Ciclev10015288m Reverse | CCCACCAACTACTATCCCCACT |
| Ciclev10021082m Forward | TTTCTTCCGCTGACAGGTTTCT |
| Ciclev10021082m Reverse | GCGTATGGTACTGGCTTCTTGC |
| Ciclev10010074m Forward | GAAGTGGAACGAGCAGAGGGAA |
| Ciclev10010074m Reverse | TCAGGGAGCAAATAAACAGAGC |
| Ciclev10010096m Forward | GCTCTGTTTATTTGCTCCCTGAT |
| Ciclev10010096m Reverse | GATGCAACTGTGGCACGGTA |
| Ciclev10032738m Forward | CTTCTTTAGTTCCTGGCTTCGTA |
| Ciclev10032738m Reverse | AAGACGGGATAGGTGCAAAATAAT |
| Ciclev10012377m Forward | AAGAACCTGGGTCATTATGCCGT |
| Ciclev10012377m Reverse | CCAATCCCCATTAGCATTCAAGTC |
| Ciclev10027736m Forward | TCAGGTATTCAGTCAGCAAGTCA |
| Ciclev10027736m Reverse | GTGCCACCATTTATAAGTGTATTA |
| Ciclev10008619m Forward | AAAGCCACGCTCCTTCTGA |
| Ciclev10008619m Reverse | CCAAAACGAGCAAACGGGT |
| Ciclev10033386m Forward | AGTGCTCCTGCTTCTCCAA |
| Ciclev10033386m Reverse | CTGCTGTAGTCTGATAAACGAAC |
| Ciclev10009779m Forward | AAATGGATGCAATACTAATCACACG |
| Ciclev10009779m Reverse | AAAGCCAGTCCGAACATAAAGC |

**Supplementary Table 3    Specific and nested PCR primers for cleavage site validation (5'-3')**

| | |
|---|---|
| Ciclev10000695m_GSP | CCAAATCATCCATTCAAATAGCG |
| Nested Ciclev10000695m_GSP | CTTCAACTTCTGAGCCACGACCT |
| Ciclev10027901m_GSP | CAACACTGTCACTGCCAGAACCC |
| Nested Ciclev10027901m_GSP | CACTTGTGAAACTACATCATCGGAAC |
| Ciclev10030860m_GSP | CGACCTACATCCTCCGATTCCA |
| Nested Ciclev10030860m_GSP | CTGGGGAAGAATGAGTTGACCG |
| Ciclev10011194m_GSP | GGCTCTGTTTCTTGGCGGTTAT |
| Nested Ciclev10011194m_GSP | TCTTTGAAGGCTGGGTTGAATGT |
| Ciclev10028090m_GSP | ATTGTAGCCATGCAAATGAAGAGG |
| Nested Ciclev10028090m_GSP | GTTTCTCATACTCTAAGATGCCAGC |
| Ciclev10011400m_GSP | GCTACATCCTTCTTCTCCAGCAAAC |
| Nested Ciclev10011400m_GSP | TTGAATCCGTGTAAATGGACTGG |
